# Supplementary material for: Deep learning-based self-supervised detection and localization of magnetic resonance imaging anomalies in artificial intelligence-supported radiotherapy workflows
Source: Phys Imaging Radiat Oncol. 2026 Oct 28;42:101114. doi: 10.1016/j.phro.2026.101114 (PMC13744269; doi:10.1016/j.phro.2026.101114)
Supplement: Supplementary file 1 [file mmc1.docx]

Supplementary material

Catching MRI outliers: unsupervised detection and localization of MRI artefacts and clinical anomalies using deep learning

Corresponding author: Mustafa Kadhim

Correspondence regarding the methods should be directed to the corresponding author. Code for the pelvic and brain MRI implementations is available in https://github.com/MustafaKadhim/Self-supervised-anomaly-detection-for-medical-images.

# Contents

1. Framework overview and design rationale
   1.1 Stage 1: residual vector-quantised variational autoencoder
   1.2 Stage 2: factorised bidirectional masked-token transformer
2. Inference and anomaly scoring
3. Data augmentation
4. Domain-specific adaptations
5. Data preprocessing and cohort splits
6. Evaluation methodology
7. Supplementary tables
8. Representative anomaly samples
9. Synthetic anomaly generation for pelvic MRI

# 1. Framework overview and design rationale

The framework was developed for unsupervised detection of out-of-distribution findings in MRI. It was trained without anomaly labels and learned representations of reference images that did not contain the evaluated anomalies. In the pelvic experiment, the reference cohort comprised prostate cancer patients from LUND-PROBE; these cases should therefore not be interpreted as healthy volunteers. In the brain experiment, reference images were obtained from healthy IXI participants and from fastMRI examinations classified as normal according to the fastMRI+ records.

The method combines two complementary principles. First, images are compressed into discrete tokens that represent coarse anatomical structure and residual texture. Second, a masked-token model learns which token configurations are expected in the reference distribution. At inference, anomaly evidence is obtained from (i) token surprisal, measured by the negative log-likelihood (NLL) of observed tokens, and (ii) perceptual differences between the input or Stage 1 reconstruction and a model-healed reconstruction. No anomaly labels or radiologist-provided bounding boxes are used for model training, calibration, threshold selection, or computation of the primary receiver operating characteristic (ROC) metric.

The framework contains two sequential stages (Figure S1):

1. **Stage 1, RVQ-VAE.** A Vision Transformer (ViT) encoder compresses each 256 x 256 MRI slice into a 32 x 32 grid of discrete tokens at two residual vector-quantisation levels. A convolutional decoder reconstructs the slice from these tokens.
2. **Stage 2, Fact-biT.** A factorised bidirectional transformer learns the distribution of tokens in the reference data by predicting masked tokens from their spatial context.

**Figure S1.** Overview of the two-stage anomaly-detection framework for MRI quality control. During self-supervised training, Stage 1 compresses each reference slice into two residual token levels (L1 and L2) and reconstructs the image; Stage 2 learns to recover masked tokens. At inference, Pathway A derives an anomaly localisation map (ALM-A) from the calibrated perceptual difference between the observed or reconstructed image and the healed image. Pathway B derives ALM-B from token surprisal estimated by repeated random masking and NLL scoring. The final binary anomaly map is formed from the union of the two pathways, with the paper-specific pelvic inpainting refinement incorporated into ALM-A.

## 1.1 Stage 1: residual vector-quantised variational autoencoder

**Figure S2.** Detailed architecture of (a) the Stage 1 RVQ-VAE and (b) the Stage 2 Fact-biT. Stage 1 comprises a ViT encoder, two residual codebook levels, and a pixel-shuffle decoder. Stage 2 performs factorised masked-token prediction, with L1 predicted from the unmasked L1 context and L2 predicted conditionally on the recovered L1 representation.

### 1.1.1 Encoder

Each single-channel 256 x 256 slice is projected into non-overlapping 8 x 8 patches by a learned convolutional embedding. The resulting 32 x 32 grid contains 1,024 tokens, each with an embedding dimension of 256. The token sequence is processed by an eight-layer ViT encoder with eight attention heads, learned positional embeddings, and an attention dropout rate of 0.1. The spatial 32 x 32 organisation is retained throughout the encoder.

### 1.1.2 Residual vector quantisation

The encoded feature map is passed through a two-level residual vector-quantisation module. L1 captures coarse structural information, whereas L2 represents residual texture information. Each level has a separately learned codebook and assigns every spatial feature vector to its nearest codebook entry by Euclidean distance.

Codebook entries are updated by exponential moving average (EMA; decay 0.85). An orthogonal regularisation term (weight 0.10) promotes codebook diversity, and infrequently activated entries are reinitialised to reduce the occurrence of inactive codes. A commitment loss (weight 0.25) encourages encoder outputs to remain close to their assigned codebook vectors. The codebook size is 192 entries per level for pelvic MRI and 256 entries per level for brain MRI.

### 1.1.3 Decoder

The decoder reconstructs the image through three pixel-shuffle upsampling blocks, each increasing the spatial resolution by a factor of two. A 512-channel stem and three 512-channel residual blocks use Group Normalisation with eight groups and SiLU activations. The three upsampling stages reduce the channel count from 512 to 256, 256 to 128, and 128 to 64. A final 64-to-1 convolution produces the reconstructed 256 x 256 grayscale image. Brain reconstructions are clamped to the normalised range [-3, 3]; pelvic reconstructions are not clamped.

The spatial quantisation error, defined by the distance between encoded features and their assigned codebook vectors, is retained as an auxiliary diagnostic output but is not added separately to the primary anomaly score.

### 1.1.4 Training objective

Stage 1 is trained end to end with

$$\mathcal{L}_{Stage 1}=\mathcal{L}_{1}+\lambda_{\mathrm{perc}}\mathcal{L}_{\mathrm{BiomedCLIP}}+\mathcal{L}_{\mathrm{commit}}.$$

The pixel reconstruction term, $\mathcal{L}_{1}$, is the mean absolute error between the input and reconstruction. The perceptual term, $\mathcal{L}_{\mathrm{BiomedCLIP}}$, is the cosine distance between L2-normalised pooled image representations from a frozen BiomedCLIP vision encoder. Images are resized to 224 x 224 and normalised using the perceptual encoder’s expected input statistics. The perceptual weight, $\lambda_{\mathrm{perc}}$, is 0.9 for pelvic MRI and 0.5 for brain MRI. The commitment term has a weight of 0.25 and uses a stop-gradient on the quantised targets.

### 1.1.5 Training protocol and inference compute

Stage 1 is trained using AdamW (β₁ = 0.9, β₂ = 0.95, weight decay 10⁻⁴) with cosine annealing over the full training duration (T_max = 100 epochs, η_min = 0). Training runs for a maximum of 100 epochs; the top three checkpoints ranked by validation loss are retained for downstream use, without early stopping. Full 32-bit precision is used throughout. Both models were trained on an Nvidia RTX PRO 6000 Blackwell GPU (96 GB GDDR7), Nvidia driver (v.570.195.03), Ubuntu (v.22.04.05), CUDA (v.12.8), and PyTorch (v.2.8.0). Further python package details are provided on our code repository. The inference time to obtain an anomaly heatmap and anomaly score was on average around 24 sec (including GPU warm-up) per pelvis case, and around 8 sec per brain case, utilizing a single Nvidia RTX PRO 6000 Blackwell GPU. This provides an initial indication of computational cost; timing will depend on hardware and inference settings.

## 1.2 Stage 2: factorised bidirectional masked-token transformer

### 1.2.1 Architecture and factorisation

Fact-biT receives the 1,024-token grid from the frozen Stage 1 encoder. A learned task embedding identifies whether L1 or L2 is being predicted. The model contains eight transformer layers, eight attention heads, a hidden dimension of 256, RMSNorm, and SwiGLU feed-forward blocks. No dropout is applied in Stage 2. Scaled dot-product attention uses Flash Attention when supported by the installed PyTorch and GPU configuration.

Prediction is factorised across the two quantisation levels. During L1 prediction, masked L1 tokens are estimated from the remaining L1 context while L2 is fully masked. L2 is then predicted conditionally on the recovered L1 representation. This separates modelling of coarse anatomical structure from residual texture while retaining a shared transformer backbone.

### 1.2.2 Positional encoding

Brain MRI uses two-dimensional rotary position embeddings (2D RoPE) over the row and column coordinates of the 32 x 32 grid, with a base frequency of 25,000. Brain slices are modelled independently; axial slice position is not used.

Pelvic MRI uses three-dimensional RoPE over row, column, and axial slice index. The maximum spatial and axial positions are 64 and 92, respectively, with a base frequency of 25,000. The axial index is parsed from the standardised slice filename and is supplied during training and inference. This allows the pelvic model to use the predictable superior-inferior organisation of prostate, bladder, rectum, and adjacent structures.

### 1.2.3 Masking and objective

Training uses an equal mixture of structured block masking and independent random masking. Block masks comprise 2 x 2 and 4 x 4 spatial regions. For random masking, the masking fraction is sampled from a Beta(4,4) distribution and clipped to [0.15, 0.75].

The Stage 2 loss is a weighted cross-entropy over the two token levels:

$$\mathcal{L}_{Stage 2}=\mathcal{L}_{\mathrm{CE}}^{\left( L1 \right)}+0.25 \mathcal{L}_{\mathrm{CE}}^{\left( L2 \right)}.$$

Label smoothing of 0.05 is applied. Stage 1 remains frozen. Stage 2 uses AdamW with $\beta_{1}=0.9$, $\beta_{2}=0.98$, and weight decay 0.01. A 2,000-step linear warm-up is followed by cosine annealing. The learning rate is ${10}^{-4}$ for pelvic MRI and $2\times{10}^{-4}$ for brain MRI. Training runs for at most 100 epochs, and the three checkpoints with the lowest validation loss are retained without early stopping. Pelvic Stage 2 training is restricted to axial slice indices 30-60; all prepared brain training slices are eligible.

# 2. Inference and anomaly scoring

Inference is performed separately for each 2D slice. Pathway A measures the perceptual change introduced when the model heals masked regions, whereas Pathway B measures how unlikely the observed L1 tokens are under the learned reference distribution.

## 2.1 Calibration on reference images

Calibration uses reference slices from the validation data only. For each calibration slice, Stage 1 produces L1 and L2 token grids, Fact-biT heals the paper-specific checkerboard masks, and the healed tokens are decoded. A spatial LPIPS map with a VGG backbone is then computed and smoothed by average pooling. The smoothing kernel is 15 pixels for pelvic MRI and 7 pixels for brain MRI.

At each pixel, the mean $\mu$ and standard deviation $\sigma$ of the smoothed LPIPS values are estimated across the calibration cases. Pelvic calibration uses 15 randomly selected validation cases. Brain calibration uses 30 mid-axial slices from normal fastMRI validation cases. Calibration and inference use identical smoothing and healing settings.

## 2.2 Pathway A: perceptual healing

Stage 1 encodes the test slice into L1 and L2 tokens. Fixed checkerboard masks are then healed by Fact-biT over six iterative prediction steps. At each step, the most confident predictions are committed until the masked positions are filled, after which Stage 1 decodes the healed token grid.

For pelvic MRI, the perceptual difference is computed between the input image $x$ and healed image $\hat{x}_{\mathrm{healed}}$. For brain MRI, it is computed between the Stage 1 reconstruction $\hat{x}$ and $\hat{x}_{\mathrm{healed}}$ to reduce sensitivity to scanner- and protocol-dependent differences between the raw image and the compressed representation. Horizontal flipping is used for test-time augmentation; the aligned maps from the original and flipped images are averaged.

The smoothed perceptual map $H$ is standardised pixelwise:

$$Z\left( u,v \right)=\frac{H\left( u,v \right)-\mu\left( u,v \right)}{\sigma\left( u,v \right)+\varepsilon}.$$

Pelvic MRI uses the one-sided criterion $Z>2.0$. Brain MRI uses the two-sided criterion $Z<-2.5$ or $Z>6.0$. The thresholded Z-score support is multiplied by the perceptual map and binarised to form ALM-A. In the pelvic implementation, targeted inpainting/backflow at the 99th percentile is incorporated as a refinement of ALM-A. This refinement is disabled in the reported brain experiment.

We calibrated a per-pixel normal reference by computing the mean (μ) and standard deviation (σ) of LPIPS values at every pixel across a randomly selected cohort of normal slices from the validation set. At inference, the perceptual difference map was converted to a Z-score map, Zscore-map=(LPIPS−μ)/(σ). The Zscore-map was thresholded (using either a one- or two-sided thresholding criterion) and binarized, producing a Zscore-map-mask that isolated pixels whose perceptual error exceeded what was normal for that anatomical location. The anomaly localization map (ALM) was obtained by multiplying the perceptual-difference map by the Zscore-map-mask, highlighting the region of the perceptual deviation. This spatial localization improves interpretability by enabling visual inspection of suspicious regions contributing to the anomaly score. Thresholding the ALM yielded the binary anomaly map A.

## 2.3 Pathway B: token surprisal

The L1 token grid is repeatedly masked at random. For every masked position $i$, Fact-biT predicts a categorical distribution over the codebook and the surprisal of the observed token $t_{i}$ is

$$s_{i}=-logp\left( t_{i}\mid\text{visible L1 context} \right).$$

Only trials in which a position is masked contribute to its estimate. The position-wise NLL is averaged over 50 trials for pelvic MRI and 100 trials for brain MRI. Pelvic inference masks 90% of L1 tokens per trial and retains NLL values greater than 8.0. Brain inference masks 80% of L1 tokens per trial and retains values greater than 5.0. The 32 x 32 token-surprisal map is bilinearly upsampled to 256 x 256 and binarised to form ALM-B.

## 2.4 Final binary map and case-level score

The final binary anomaly map is the logical union of ALM-A and ALM-B:

$$M_{\mathrm{final}}=M_{A}\cup M_{B}.$$

The pelvic inpainting/backflow refinement, when present, is included within $M_{A}$ and is not counted as a separate score. Similarly, auxiliary token-surprisal counts written by the code are not added again, because their binary support is already contained in $M_{B}$.

For the pelvic evaluation, the score for patient $p$ is the sum of detected pixels over the 12 central slices with indices 38-49:

$$S_{p}=\sum_{z=38}^{49} \sum_{u,v} M_{\mathrm{final}}^{\left( p,z \right)}\left( u,v \right).$$

For the brain evaluation, one representative axial slice is evaluated per included patient/anomaly category. For local findings, this is the slice containing the largest fastMRI+ bounding-box annotation. The brain case score is therefore the score from that single selected slice. Global findings do not have bounding boxes and are evaluated using their selected representative slice and study-level label. This distinction is important: the brain ROC analysis does not aggregate scores over a full MRI volume.

# 3. Data augmentation

Augmentation is applied only during training. Data-loader and model-internal augmentation modules are distinct; the brain training workflow uses the data-loader pipeline as the primary source and disables duplicate model-internal transforms when needed to avoid applying the same operation twice.

## 3.1 Brain MRI

The brain cohort contains substantial scanner- and protocol-dependent variability. The training pipeline therefore applies broader augmentation as presented in Table S6.

## 3.2 Pelvic MRI

LUND-PROBE was acquired using a standardised institutional protocol. Pelvic augmentation is therefore more conservative. The model-internal module applies random intensity scaling (probability 0.33) and an affine transform with rotation up to $\pm5^{\circ}$ and horizontal translation up to $\pm5$ pixels (probability 0.33). When data-loader augmentation is enabled, it adds a horizontal flip (probability 0.50) and rotation up to $\pm5^{\circ}$ (probability 0.30). No pelvic contrast, Gaussian-noise, or zoom augmentation is applied during model training.

# 4. Domain-specific adaptations

The pelvic and brain implementations share the same two-stage design and final scoring principle, but several settings are adapted to the acquisition characteristics of each domain.

## 4.1 Token representation and positional information

The pelvic model uses 192 entries per codebook level and 3D RoPE. The brain model uses 256 entries per level and 2D RoPE. Pelvic slice indices are required because the transformer conditions on axial position; the brain transformer treats each slice independently and ignores slice position.

## 4.2 Perceptual reference and reconstruction range

Pelvic LPIPS compares the input with its healed version. Brain LPIPS compares the Stage 1 reconstruction with its healed version. Brain decoder outputs are clamped to [-3, 3], matching the brain preprocessing range; pelvic outputs are not clamped. Calibration maps are therefore domain specific and are not interchangeable.

## 4.3 Healing and surprisal settings

Pelvic healing uses two complementary 2 x 2 checkerboard patterns, a healing temperature of 0.3, and an inpainting temperature of 0.3. Brain healing uses the reported 4 x 4 checkerboard configuration, a healing temperature of 0.9, and an inpainting temperature of 0.5. Brain token surprisal uses more Monte Carlo trials (100 vs 50), whereas pelvic inference masks a larger fraction of tokens in each trial (0.90 vs 0.80).

## 4.4 Slice use

Pelvic Stage 2 training is restricted to indices 30-60, while the reported pelvic evaluation uses the 12 central slices 38-49. IXI preprocessing retains axial slices 128-188 for training. Prepared fastMRI slices are treated independently; their original slice indices are not used by the brain transformer’s positional encoding. The brain evaluation uses one representative slice per patient/anomaly category, as described in Section 2.4.

# 5. Data preprocessing and cohort splits

All data partitions are defined at the patient level before model fitting or evaluation. No patient appears in more than one training, validation, normal-test, or anomaly-test partition.

## 5.1 Pelvic MRI: LUND-PROBE and clinical cohorts

LUND-PROBE comprises T2-weighted pelvic MRI from 467 prostate cancer patients treated in an MRI-only radiotherapy workflow at Skane University Hospital, Lund, Sweden. Images were acquired on a single 3 T GE Healthcare scanner using the clinical protocol, with the scan centred around the pubic bone. The cohort was split into training ($n=384$), validation ($n=29$), and test ($n=54$) sets.

Five test patients with hydrogel rectal spacers were removed from the reference test subset and treated as clinical anomalies. The remaining 49 anomaly-free test patients served as the unmodified reference cases and as the source images for the five synthetic global artefact categories. The Fourier-spike simulation produced an insufficiently visible perturbation in three cases; these were excluded from that category, leaving 46 spike cases. The synthetic cohort therefore comprised 242 cases: motion ($n=49$), noise ($n=49$), ghosting ($n=49$), Fourier-space spikes ($n=46$), and blur ($n=49$).

The clinical pelvic anomaly cohort comprised 36 cases: the five LUND-PROBE spacer cases and 31 retrospective or volunteer examinations. The latter included hip implants acquired with the LUND-PROBE protocol ($n=4$) or different protocols ($n=4$), cervical brachytherapy applicators ($n=4$), cropped fields of view ($n=14$), and five volunteer scans with global acquisition or protocol variations (T1-weighted, proton-density-weighted, noise, motion, and ghosting; one case each). One additional volunteer scan without an anomaly served as a normal comparison.

Raw pelvic volumes are Z-score normalised per volume. Axial slices are saved as float32 arrays using a filename that preserves the patient identifier and zero-padded axial index. During loading, slices are rotated 90 degrees clockwise, resized to 320 x 320 using area interpolation, and centre-cropped to 256 x 256.

## 5.2 Brain MRI: IXI, fastMRI, and fastMRI+ annotations

The brain experiment uses images from IXI and fastMRI. fastMRI+ supplies radiologist annotations linked to fastMRI examinations; it is not treated as a separate source of image volumes.

IXI contributes 581 T1-weighted examinations from healthy participants acquired at three hospitals in London, United Kingdom, and is used only for model development. Volumes are reoriented to the closest canonical orientation, Z-score normalised per volume, clipped to [-3, 3], and restricted to axial indices 128-188. Slices are rotated 90 degrees counter-clockwise, centre-cropped or padded to 256 x 256, and stored as float32 arrays.

Using the fastMRI+ global and bounding-box records, 172 fastMRI examinations were classified as normal and 255 as anomalous. The normal fastMRI examinations were split into training ($n=112$), validation ($n=30$), and test ($n=30$) sets. Examinations were acquired at 1.5 T or 3 T using heterogeneous protocols. A subgroup labelled as small-vessel chronic white-matter ischaemic change was excluded because bounding-box annotations were unavailable and reliable visual localisation could not be established for the planned representative-slice evaluation.

For local fastMRI+ findings, the bounding boxes are stored only to identify the representative slice and to overlay the radiologist annotation during qualitative review. They are not used to train or calibrate either model stage and do not enter the primary ROC calculation.

# 6. Evaluation methodology

## 6.1 ROC analysis

The unit of analysis is the patient/case. Pelvic patients contribute one score aggregated over the 12 predefined central slices. Brain patients contribute one score from the predefined representative slice. Reference cases are assigned label 0 and anomaly cases label 1. The ROC curve is calculated across the full range of score thresholds, and its area under the curve (AUC) is the primary discrimination metric.

## 6.2 Confidence intervals

Uncertainty is estimated using a non-parametric stratified bootstrap at patient level. Positive and negative cases are sampled separately with replacement for 2,000 replicates. The 95% confidence interval for the AUC is the 2.5th to 97.5th percentile interval. A pointwise 95% ROC confidence band is obtained on a fixed grid of 201 false-positive-rate values.

## 6.3 Operating-point and category analyses

Sensitivity and specificity are reported at the prespecified 5% false-positive-rate operating point. Pelvic results are also stratified by synthetic artefact category and by clinical anomaly group. Brain anomalies are grouped according to the fastMRI+ global and local labels. These labels are used only after inference for evaluation and presentation.

## 6.4 Precision-recall analysis

For pelvic MRI, area under the precision-recall curve (AUPRC) is calculated as a secondary metric using right-step integration. The brain analysis reports ROC AUC as its primary quantitative metric.

## 6.5 Use of fastMRI+ bounding boxes

No quantitative bounding-box overlap, pixel precision, F1 score, or box-recall metric is reported in the study. For local findings, the largest bounding box is used to select one representative axial slice. Bounding boxes are subsequently overlaid on the anomaly heatmaps as a qualitative sanity check of spatial correspondence. Global labels, including motion and extra-axial collection, do not have local bounding boxes and are reviewed using the spatial distribution of the heatmap alone.

# 7. Supplementary tables

**Table S1. Stage 1 architecture and training settings.**

| **Setting** | **Pelvic MRI** | **Brain MRI** |
| --- | --- | --- |
| Input resolution | 256 x 256 | 256 x 256 |
| Patch size | 8 x 8 | 8 x 8 |
| Token grid | 32 x 32 (1,024 tokens) | 32 x 32 (1,024 tokens) |
| Encoder depth / heads | 8 / 8 | 8 / 8 |
| Embedding dimension | 256 | 256 |
| Encoder attention dropout | 0.1 | 0.1 |
| RVQ levels | 2 | 2 |
| Codebook entries per level | 192 | 256 |
| EMA decay | 0.85 | 0.85 |
| Orthogonal regularisation weight | 0.10 | 0.10 |
| Commitment weight | 0.25 | 0.25 |
| Decoder channels | 512 -> 256 -> 128 -> 64 -> 1 | 512 -> 256 -> 128 -> 64 -> 1 |
| Decoder output clamp | None | [-3, 3] |
| Reconstruction loss | L1 | L1 |
| Frozen perceptual encoder | BiomedCLIP ViT | BiomedCLIP ViT |
| Perceptual weight | 0.9 | 0.5 |
| Optimiser | AdamW | AdamW |
| Learning rate | $1\times{10}^{-4}$ | $2\times{10}^{-4}$ |
| $\beta_{1},\beta_{2}$ | 0.9, 0.95 | 0.9, 0.95 |
| Weight decay | $1\times{10}^{-4}$ | $1\times{10}^{-4}$ |
| Maximum epochs | 100 | 100 |
| Batch size | 128 | 192 |
| Precision | 32-bit | 32-bit |
| Checkpoint selection | Three lowest validation losses | Three lowest validation losses |

**Table S2. Stage 2 architecture and training settings.**

| **Setting** | **Pelvic MRI** | **Brain MRI** |
| --- | --- | --- |
| Positional encoding | 3D RoPE (row, column, slice) | 2D RoPE (row, column) |
| RoPE base | 25,000 | 25,000 |
| Maximum position | 64 spatial; 92 axial | 33 spatial |
| Transformer depth / heads | 8 / 8 | 8 / 8 |
| Embedding dimension | 256 | 256 |
| Normalisation / activation | RMSNorm / SwiGLU | RMSNorm / SwiGLU |
| Transformer dropout | None | None |
| Masking mixture | 50% block; 50% random | 50% block; 50% random |
| Random masking distribution | Beta(4,4), clipped [0.15, 0.75] | Beta(4,4), clipped [0.15, 0.75] |
| L1 / L2 cross-entropy weights | 1.0 / 0.25 | 1.0 / 0.25 |
| Label smoothing | 0.05 | 0.05 |
| Optimiser | AdamW | AdamW |
| Learning rate | $1\times{10}^{-4}$ | $2\times{10}^{-4}$ |
| $\beta_{1},\beta_{2}$ | 0.9, 0.98 | 0.9, 0.98 |
| Weight decay | 0.01 | 0.01 |
| Warm-up | 2,000 steps | 2,000 steps |
| Maximum epochs | 100 | 100 |
| Batch size | 128 | 158 |
| Stage 1 during training | Frozen | Frozen |
| Eligible training slices | Axial indices 30-60 | All prepared slices |

**Table S3. Reported inference settings.**

| **Setting** | **Pelvic MRI** | **Brain MRI** |
| --- | --- | --- |
| LPIPS reference | Input vs healed | Reconstruction vs healed |
| Calibration cases | 15 validation patients | 30 normal validation slices |
| Smoothing kernel | 15 pixels | 7 pixels |
| Checkerboard configuration | Two complementary 2 x 2 patterns | 4 x 4 configuration |
| Healing steps | 6 | 6 |
| Healing temperature | 0.3 | 0.9 |
| Inpainting temperature | 0.3 | 0.5 |
| Test-time augmentation | Horizontal flip; maps averaged | Horizontal flip; maps averaged |
| Z-score criterion | $Z>2.0$ | $Z<-2.5$ or $Z>6.0$ |
| Inpainting/backflow refinement | Enabled at 99th percentile | Disabled |
| Token-surprisal trials | 50 | 100 |
| Token mask fraction | 0.90 | 0.80 |
| NLL retention threshold | 8.0 | 5.0 |
| Evaluation slices per case | 12 (indices 38-49) | One representative slice |

**Table S4. Cohort and split overview.**

| **Cohort component** | **Pelvic MRI** | **Brain MRI** |
| --- | --- | --- |
| Reference image source | LUND-PROBE prostate cancer patients | IXI healthy participants and normal fastMRI examinations |
| Image weighting | T2-weighted | T1-weighted |
| Development split | 384 training; 29 validation | IXI: 581 development only; normal fastMRI: 112 training, 30 validation |
| Normal test cases | 49 anomaly-free LUND-PROBE patients | 30 normal fastMRI patients |
| Anomaly test cases | 242 synthetic and 36 clinical | 255 fastMRI patients identified from fastMRI+ records |
| Synthetic categories | Motion, noise, ghosting, Fourier-space spikes, blur | Not applicable |
| Clinical/annotated categories | Spacer, hip implant, brachytherapy applicator, cropped FOV, protocol/acquisition variations | Global and local fastMRI+ labels |
| Primary evaluation unit | Patient score over 12 central slices | Patient score from one representative slice |
| Bounding-box role | Not available | Local-slice selection and qualitative overlay only |

**Table S5. Principal domain-specific differences.**

| **Design choice** | **Pelvic MRI** | **Brain MRI** | **Consequence** |
| --- | --- | --- | --- |
| Codebook size | 192 per level | 256 per level | Checkpoints and token distributions are domain specific |
| Positional encoding | 3D RoPE | 2D RoPE | Pelvic inference requires a valid axial index; brain inference is independent of slice position |
| Decoder range | Unclamped | Clamped to [-3, 3] | Calibration maps cannot be exchanged between domains |
| LPIPS reference | Input | Stage 1 reconstruction | The perceptual maps have different semantics |
| Perceptual Z threshold | One-sided | Two-sided | Domain-specific calibration of unusually high or low discrepancy |
| Token-surprisal trials | 50 | 100 | More sampling is used for heterogeneous brain data |
| Token mask fraction | 0.90 | 0.80 | Different visible-context fractions during inference |
| Evaluation aggregation | 12 slices | One selected slice | Both yield one score per patient, but by different aggregation rules |

**Table S6. Brain image augmentations.**

| **Transform** | **Parameters** |
| --- | --- |
| Random intensity scaling | Factor $\pm0.10$, probability 0.33 |
| Random contrast | $\gamma\in\left[ 0.5,1.5 \right]$, probability 0.33 |
| Gaussian noise | Standard deviation 0.30, probability 0.50 |
| Affine rotation | $\pm{15}^{\circ}$, probability 0.33 |
| Affine translation | $\pm15$ pixels along both axes, probability 0.33 |
| Affine zoom | Scale 0.80-1.20, probability 0.33 |
| Horizontal flip | Probability 0.50 |

# 8. Representative anomaly samples

**Figure S3.** Representative pelvic MRI cases included in the reported study. * a synthetic global artefact generated from an anomaly-free LUND-PROBE test case. ** volunteer acquisitions with normal appearance or global acquisition/protocol variations. *** clinical cases acquired with a T2-weighted protocol that differed from the LUND-PROBE protocol. The hydrogel spacer and the hip implant acquired with the LUND-PROBE protocol are shown without an asterisk. FOV, field of view; PD, proton density.

**Figure S4.** Representative T1-weighted fastMRI examinations from the normal and anomalous cohorts. The category labels follow the linked fastMRI+ radiologist records. fastMRI provides the image volumes; fastMRI+ provides the global labels and local bounding-box annotations used for cohort selection and qualitative review. No bounding-boxes are used as overly for this figure.

# 9. Synthetic anomaly generation for pelvic MRI

Five global synthetic artefact categories were generated from the 49 anomaly-free LUND-PROBE test volumes: rigid motion, Gaussian noise, ghosting, Fourier-space spikes, and whole-volume Gaussian blur. Each transformation was applied in three dimensions before the downstream slice preprocessing described in Section 5.1. An unmodified copy of each source volume served as its reference case and was not counted as an anomaly category.

Randomness is intrinsic to the motion, noise, ghosting, and spike transforms. The whole-volume blur is deterministic for a specified kernel. The code and exact experiment configuration are available in the project repository.

## 9.1 Software

## Table S7. Used software packages

| **Package** | **Role** |
| --- | --- |
| TorchIO | Three-dimensional MRI motion, spike, ghosting, and noise simulation |
| nibabel | NIfTI input and output |
| SciPy | Three-dimensional Gaussian filtering |
| NumPy | Array handling and reproducible random-state management |
| joblib | Parallel processing across patients |

## 9.2 Synthetic artefact categories

### 9.2.1 Rigid motion

TorchIO RandomMotion simulates rigid-body rotation and translation at multiple time points during acquisition and reconstructs the perturbed volume with linear interpolation. Maximum rotations are $\pm{30}^{\circ}$, maximum translations are $\pm50$ mm, and 10 motion events are simulated. The transformation probability is 1.0.

### 9.2.2 Fourier-space spikes

TorchIO RandomSpike inserts seven impulses at randomly selected frequency-space locations with an intensity factor of 0.95 relative to the frequency-space magnitude. The transformation probability is 1.0. Three generated cases were excluded because the random perturbation was too small to produce a discernible artefact, leaving 46 cases in this category.

### 9.2.3 Ghosting

TorchIO RandomGhosting generates two to four shifted ghost copies along the configured phase-encoding axes (axes 0 and 1). The ghost intensity factor is 2.0 and the restoration factor is 0.02. The transformation probability is 1.0.

### 9.2.4 Gaussian noise

TorchIO RandomNoise adds independent Gaussian noise throughout the volume. The experiment configuration uses a mean of 2,000 and standard deviation of 600 in the raw image-intensity units. The transformation probability is 1.0.

### 9.2.5 Whole-volume Gaussian blur

A three-dimensional anisotropic Gaussian filter is applied to the complete volume using standard deviations of 6 voxels in each in-plane direction and 2 voxels through plane. This simulates a global reduction in spatial resolution.

## 9.3 Processing and outputs

Transformations are applied across patients using joblib.Parallel. Each output is saved as a compressed NIfTI file in a subdirectory corresponding to its transformation type. Patient identifiers remain linked to the source case to allow paired processing and auditing, while all train/validation/test boundaries remain unchanged.

## 9.4 Summary of simulated artefacts

**Table S8. Synthetic pelvis image augmentations.**

| **Reported category** | **Cases** | **Scope** | **Implementation** | **Key settings** |
| --- | --- | --- | --- | --- |
| Rigid motion | 49 | Whole volume | TorchIO RandomMotion | $\pm{30}^{\circ}$; $\pm50$ mm; 10 events |
| Gaussian noise | 49 | Whole volume | TorchIO RandomNoise | Mean 2,000; SD 600 |
| Ghosting | 49 | Whole volume | TorchIO RandomGhosting | 2-4 ghosts; intensity 2.0; restoration 0.02 |
| Fourier-space spikes | 46 | Whole volume | TorchIO RandomSpike | 7 spikes; intensity 0.95 |
| Gaussian blur | 49 | Whole volume | SciPy Gaussian filter | $\sigma=\left( 6,6,2 \right)$ voxels |

The total synthetic anomaly cohort contains 242 cases. Only the five global artefact categories summarised above contribute to the reported evaluation.
